# Asymmetric Extraction Decision in Orthodontics

**DOI:** 10.7759/cureus.40162

**Published:** 2023-06-08

**Authors:** Shalabh Baxi, Virag Bhatia, Anand A Tripathi, Pratiksha Kumar, Anil Pandey, Pradeep Dilip Taide

**Affiliations:** 1 Department of Orthodontics, Government Dental College, Raipur, IND; 2 Department of Orthodontics, Government College of Dentistry, Indore, IND; 3 Department of Orthodontics, Saraswati Dhanwantari Dental College and Hospital, Parbhani, IND; 4 Department of Oral Pathology and Microbiology, Government College of Dentistry, Indore, IND; 5 Department of Pedodontics and Preventive Dentistry, Government Dental College, Raipur, IND; 6 Department of Prosthodontics, Yogita Dental College and Hospital, Khed, IND

**Keywords:** second molar extraction, first molar extraction, premolar extraction, incisor extraction, factors affecting choice of extraction, asymmetric extraction decision, dental asymmetry

## Abstract

During the treatment planning process, extraction decisions are crucial. As a therapy option, the extraction of teeth should be considered in instances where there is a lack of facial harmony and stability in the occlusion. Treatment aims, the kind of malocclusion, aesthetic considerations, and growth patterns are all factors that influence asymmetric extraction. For the most part, premolar extractions are required when there is a significant midline difference or an asymmetrical connection between the teeth. Premolars, which are the first teeth to erupt and occupy the posterior position in chewing, are more vulnerable to injury than other permanent teeth. The optimal time to remove a second molar is at the moment when the connection between the molars has normalized or when a major front cross-bite can be remedied.

## Introduction and background

Orthodontic treatment's primary objective is to restore the teeth's natural connection with the surrounding facial features. Edward H. Angle emphasized the preservation of all dental elements for the benefit of facial symmetry [1]. Extraction of a tooth removes the potential of establishing an ideal occlusion or ideal aesthetics, according to his reasoning. However, while correcting the malocclusion orthodontically, the facial balance may become worse. This could be due to ignored soft tissue relationships during diagnosis or a lack of attention to aesthetic goals.

So the current trend in orthodontics is towards soft tissue relationships. Proffit et al. explained that this change in treatment goals towards soft tissues and away from dental and skeletal relations represents the paradigm shift [1]. It was not always feasible to keep all of the dental components in order to rectify certain types of malocclusions. In order to address some forms of malocclusion, further investigations on the treatment's stability showed that tooth extractions were necessary. Patients with dental position asymmetries can be certain they will have a functional bite after treatment with this management [1].

## Review

Dental asymmetry

It is impossible to find complete symmetry in nature. Facial and dental asymmetry is a typical finding when contrasting the right and left sides of the body. According to Lundstrom, the asymmetry of the arches may be categorized quantitatively or qualitatively [2]. Quantification asymmetries include asymmetrical tooth counts on each side of an arch as well. In terms of dental arch connections to the skull and to each other, these differences in tooth size and location would be classified as qualitative asymmetries. When it comes to correcting quantitative asymmetries, options like prosthetic rehabilitation or fixed orthodontics are viable options. A novel strategy for dealing with qualitative dental asymmetry is to remove a group of teeth in order to simplify the mechanics of the inter- and intra-archs. When it comes to addressing qualitative asymmetries, the best solutions are stripping, single-incisor extraction, and asymmetrical permanent tooth extraction, followed by asymmetrical mechanics [2].

Tooth size asymmetry was studied by Black as early as 1902 [3]. In 1956, Murray revisited this issue from a slightly different perspective [4]. He studied the breadth-size relationship between six maxillary and mandibular anterior teeth in 400 treated orthodontic cases and found that more than 50% of the mandibular anterior teeth were 2 mm or larger than required for the ideal tooth-to-tooth and arch-to-arch relationships. It was his conclusion that this variation in tooth size and the relationship between upper and lower anterior segments are of sufficient incidence and magnitude to merit serious consideration in the aetiology, diagnosis, and treatment planning of every orthodontic case. In 1994, Murray measured the teeth on 500 sets of study models [5]. He concluded that 90% of the cases had left-right width discrepancies in one or more pairs of contralateral teeth, a condition prejudicial to perfect balance. Bolton analyzed 55 patients with excellent occlusion to determine if mathematical ratios could be established between the total lengths of dental arches to aid in diagnosis and treatment planning. He noted that regardless of mathematical findings, each patient required individual consideration. The numbers arrived at were useful only as an aid in proportional deviation; however, an alteration of tooth dimension in one of the arches had to be considered. This alteration could range from dental re-proximation to extraction [6].

The asymmetric extraction decision

In order to rectify the midline deviation, unilateral movement of the posterior teeth, and crossbite, asymmetric extractions would be necessary and important. It is important to reduce the amount of time needed for treatment and tooth mobility to simplify the mechanics of orthodontics and get results that are more stable and functional. Treatment options for facial balance and occlusion stability may require the excision of teeth if there are significant quality differences. The process of removing teeth is still a problem, particularly when it comes to deciding which tooth should be removed [3]. The choice to extract teeth for orthodontic treatment should not only be based on the amount of space between the teeth but also on other factors, such as the appearance and the stability of the face. Through the use of preventive extraction, it is possible to avoid the external root resorption of the second molar that is caused by an impacted molar. Cone-beam computed tomography (CBCT) allows for improved diagnostics, which leads to the selection of the optimal course of action [4]. The treatment differs depending on the degree to which resorption has occurred. When a permanent molar displays extensive root resorption, an impacted third molar can successfully substitute the tooth by moving mesially with the use of an appropriate orthodontic treatment. This is done by moving the tooth into a more favourable position. If a supernumerary premolar develops at a later time, you should have periodic panoramic X-rays taken to check for any pathological changes. If the patient has a history of having a supernumerary tooth in the front area of their mouth, there is a 24% probability that they may get an additional supernumerary premolar at a later time [2,4].

Factors affecting the choice of extraction are treatment objectives, type of malocclusion, aesthetics (large chin button, prominent nose), growth pattern, conditions of teeth (caries, multifilled teeth, impacted, ectopic, severe rotation), and health of supporting tissues [3]. According to Rheude et al., asymmetrical extractions may only be performed using plaster casts for orthodontic treatment planning. The diagnostic procedures that were used to establish the treatment plans were: Bolton's tooth size analysis, space available/space needed assessment, and a laboratory diagnostic set-up [4].

Incisor extractions

As long as there is an anterior Bolton disparity of more than 5 mm, the mandibular incisors may be extracted. This is because the anterior discrepancy is a direct indication of extraction. Mandibular incisor extractions are recommended when there is more mandibular anterior tooth material than maxillary anteriors, according to Riedel et al., who also mention this as a possible criterion [7].

Mandibular incisor extractions

In cases of moderate to severe crowding, the extraction of a single incisor will preserve the arch shape without affecting inter-canine width. Good torque control and accurate tracking of the mandibular teeth's axial tilt will help avoid lingual tilting of the mandibular cuspid, which in turn will help to keep the inter-canine width constant. For a moderate class III instance or an edge-to-edge bite, removing the lower incisors might be useful [6].

Maxillary incisor extractions

Upper central incisor extractions in orthodontics are quite rare. In the event that an upper central incisor has a bad prognosis because of deformity or because it has been severely deteriorated and is in an irreparable state, we must be prepared for the worst. Maxillary central incisor extraction will be influenced by factors such as the amount of space required and the shape, size, and root height of the adjacent lateral incisors and canines. The removal of a maxillary lateral incisor is most often performed for this purpose [3].

Cuspid extractions

These two teeth are referred to as the cornerstones of the dentition. Maxillary cuspids have not been extensively studied in terms of their aesthetic value in the scholarly literature [1]. When the affected maxillary cuspids are at an unfavourable angulation to be brought into alignment, there are clear symptoms of extraction. In these circumstances, the premolar's pre-existing inclination should be sufficient to generate an aesthetic grin arc in the patient while replacing the cuspid. If a cuspid is positioned ectopically, it will suffer from loss of attachment, which can only be remedied by transferring it to a more stable location in the jaw [8].

Premolar extractions

It is not always a simple task to provide an accurate diagnosis in orthodontic cases with Angle Class I malocclusion that necessitate the excision of the first or second premolars. This is especially true in the wide group of instances that are known as borderline situations. When planning an operation that involves the removal of the first and second premolars, it is imperative that certain characteristics, which are regarded as essential components of a diagnosis, be thoroughly evaluated. These features include a mismatch between the teeth and the alveolar bone, interactions between the maxilla and mandible, the facial profile and pattern, skeletal development, tooth asymmetries, illnesses, and patient cooperation. However, depending on the specifics of the case, a single factor might be sufficient to decide whether the first or second set of premolars should be removed. Premolar extractions are typically required when a patient has significant midline discrepancies or a disproportionate molar relationship. There should be enough room for the extraction of the first premolars in order to deal with orthodontic tooth development and biomechanics for the arrangement and proper withdrawal of teeth [9]. Gianelly et al. claim that removing the early premolars would simplify space maintenance while also preserving the point of contact between the subsequent premolar and the molar [10].

One-sided extraction of the second premolars may be advantageous, but depending on the amount of area of curve length inaccuracy, it is possible to remove the first premolars from one side and the second premolars from the other, as Vanden demonstrated [8]. The removal of the second and first premolars is an effective treatment option for patients who have class II malocclusions and have lower incisors that are moderately enlarged or bulge out of their sockets. To treat the protruding upper front teeth (if existent) and to correct any modest bulge or swarm in the lower front teeth, this method is preferred since it has little to no facial impact [9,10]. More anchoring loss in the mandible would be required to rectify the molar relationship. Class II elastics, used in conjunction with a facebow or a trans-palatal arch, might make this procedure easier and prevent the loss of the maxillary anchoring. It may be feasible to remove a first premolar on one side and a second premolar on the other; however, this will depend on the location of the arch discrepancy [11,12].

At this time, the class II subdivision malocclusions that have a lower dental midline deviation are the cases of asymmetric extractions that are discussed and argued about the most frequently. Under these conditions, either a symmetrical extraction of all four premolars or an asymmetrical extraction of three premolars is the best course of action to consider. Because of the need for intermaxillary elastics, the first alternative will only operate with the participation of the patient. When just one lower premolar (opposite to the midline deviation) is extracted, the treatment process results in an asymmetrical molar relationship (classes I and II). As a result, midline correction and improved vertical control would be more straightforward in these scenarios. In class II subdivision instances, a three-premolar extraction strategy reduces treatment time and provides better outcomes [13,14].

First molar extraction

First molars are the most vulnerable teeth because they are located at the rear of the mouth, where mastication takes place, and they are the most durable teeth. Da Silva Campos et al. believe that in order to address the problem of vertical growth and attain a class I molar relationship at the treatment's end, molar extractions are appropriate and necessary, respectively. However, seeing the third molars in the jaw and their location before extraction is a huge relief. Endodontically or periodontally impaired first molars may need to be extracted in a scenario where a large curve space is needed and the present first premolars are otherwise healthy. Because of the lower second molar's mesio-lingual inclination, therapy takes a long time when this occurs [12]. Sandler et al. have emphasized the clinical significance of primary molar extraction, which triggers mesial growth of the second and third molars and, as a result, causes a counter-clockwise mandibular turn at the end of the mandibular plan [15].

Second molar extraction

Second molar extractions should be performed as soon as possible in order to normalize the molar relationship or to correct a severe front crossbite, for as long as there are third molars back to them. However, the extraction of lower posteriors is not advised if it is difficult to close the extraction space, such as in horizontal growers with strong facial musculature [16].

Extraction protocols for class III subdivision cases

To put it another way, let us assume that the patient with class III area malocclusion has connections in his or her teeth that are both class I and class III.

Class III subdivision with proclination of upper and lower anteriors

The patient has a region of the brain classified as class III. For patients with curved profiles who have malocclusions characterized by proclined maxillary and mandibular dentitions, extractions of both the mandibular and maxillary first premolars, as well as the maxillary premolar on the class I side, are necessary to correct the midline deviation. You may use this without affecting how the teeth are connected to each other [17].

Class III subdivision with mandibular midline deviation

The extraction of the long-lasting mandibular first molar on the class III side, followed by the withdrawal of premolars and canines, and the protraction of the super-durable mandibular second molar, is recommended for patients with class III region malocclusions, as long as the patient has a straight profile [16]. In order to correct the midline deviation and front cross chomp, the teeth in front of the molar are withdrawn and the second molar is protracted in order to complete the class I molar connection. However, a normal molar connection is maintained even after undergoing the identical extraction procedure on the class III side of the mouth [18,19].

Mandibular dental midline deviation

Mandibular molars are distal on the class II side; the canine is distal; and the maxillary midline is surprisingly located with the facial midline on this side. If the patient's maxillary dental midline matches their facial midline, then extraction of the third molars could be the best option. Class I mandibular premolar removal creates a canine that is separated from the contralateral canine. The midline harmony of the face would be maintained if two upper premolars were removed [11]. After removing the class II molar on that side, the remaining three buccal parts may be fitted with intermaxillary elastics. To address all class II side extraction gaps, the maxillary front teeth and the distal mandibular component must be entirely prolonged if a mandibular premolar on the class II side must be detached. It is possible that removing a maxillary first premolar and a mandibular second premolar as part of a class II differential extraction technique will help to reduce damage in the lower teeth [11].

Maxillary dental midline deviation

In class II area malocclusions, deviation of the maxillary dental midline from the facial midline may be caused by a variety of factors, including the premature loss of a crucial maxillary second molar, which causes the long-lasting first molar on the class II side to float mesially. For instance, one patient was found to have strayed to the right of the facial midline while being treated for left malocclusion of class II development. An extraction of one of the class II premolars on the class II side would greatly aid in the treatment of a mandibular curve that does not need extractions. In order to manage the molar port, a latent or partially enacted trans-palatal curve may be used, and a space conclusion could be reached mainly using class I mechanics with little patient consistency [11].

Maxillary and mandibular dental midline deviation with skeletal symmetry

People with maxillary and mandibular medians that deviate from the facial median are very rare. Premolar extractions on opposite sides, if possible, would be preferable. How much midline therapy is desired and the major buccal segment relationship determine whether the first or second premolars should be removed. Lopsided bend swarming occurs when the maxillary and mandibular midlines are both off from the face midline, but in this condition they are on opposite sides of one another. A more appropriate arrangement under these circumstances could be to remove a class II upper premolar and a class III mandibular premolar, provided the buccal fragments reflect this imbalance. In unbalanced malocclusions, these space conclusion controls should not be discernible on either side of the dental curve. It is necessary for the model to have one kind of C-space conclusion and one sort of A-space conclusion [11]. Treatment goals, including midline revision, proper anteroposterior tooth positioning, improving the facial profile, and ensuring dental stability, all require these mechanical contrasts. As previously stated, extractions that are not properly balanced might result in an off-kilter molar position. Both sides of the mouth might have a class II or III connection with the final molar [11].

The cephalometrics of class II examples were the focus of the research by Janson et al. [18]. Patients with class II area malocclusions who had had orthodontic treatment as a result of asymmetrical and awry extractions were studied cephalometrically. It includes 54 individuals with class II development malocclusion and complete supplementation of long-lasting teeth, including first molars, at the beginning of therapy. Twenty-seven patients from group 1 were given the uneven extraction of three as their treatment. In group 2 (n = 27), four premolars were extracted symmetrically. Both pre- and post-treatment lateral cephalometric radiographs were recorded. According to the results, there were more noticeable expansions of the mandibular ejection of the incisor and of the mandibular molar imbalance file among those who had wrong extractions than among those who had symmetric extractions. Class II region malocclusions are thought to have a lower risk of incisor and delicate tissue withdrawal if the three-premolar uneven extraction convention is used [20].

Treatment of class II area malocclusions with symmetric and awry extractions was examined for its effectiveness in this review [21]. He chose 71 individuals with full class II area malocclusion as a case study and presented it. Patients in group 1 had four premolar extractions. Thirty-one individuals in group 2 had their third premolars removed (two maxillary premolars and one mandibular premolar on the class I side). Dental projects were evaluated using the Peer Assessment Rating Occlusal Index to determine the effectiveness of each treatment method. The clinical graphs were used to calculate the length of time spent in therapy. In addition, we looked at the initial and final midline deviation measurements as well as the improvement in midline deviation treatment. The rate of occlusal improvement divided by the treatment period was used to calculate efficiency. The findings revealed that group 2 had a smaller final midline deviation measure and a larger revision of the midline deviation measure than group 1 [22]. The success rate of treating type 1 and class II area malocclusions with three or four premolar extractions is comparable. Treatment including the removal of three premolars, on the other hand, results in a higher success rate for occlusal closure [19].

Janson et al. [18] undertook a cephalometric investigation. The purpose of this study is to do a cephalometric analysis of the dentoskeletal and soft tissue alterations caused by class II area malocclusion therapy using one and three premolar extraction protocols. A total of 63 patients were selected for his study and categorized into two categories. Class II malocclusions developing in 31 individuals were treated with incorrect extractions of two upper maxillary premolars and one lower premolar on the class I side of their mouth. One maxillary first premolar was unequally extracted from each of the 32 patients in group 2, all of whom had class II area malocclusion. Group 1 experienced a more pronounced reduction in maxillomandibular sagittal error and a more pronounced expulsion of maxillary first molars, the lingual inclination of mandibular incisors, and the retraction of mandibular incisors. There was mandibular inclination and protrusion in the second group. There are substantial differences between these two standards for class II developmental malocclusions of kinds 1 and 2 that warrant a distinct approach to treatment [23].

A study performed by Irfan and Fida examined the effects of uneven and symmetrical extraction designs on soft and hard tissue. It is a cross-sectional review, as the name implies [24]. Symmetric extraction patterns (SEP) were referenced by bunch 1, whereas awry extraction designs (AED) were respected by bunch 2. This study's findings demonstrate that there was a significant difference between pre- and post-treatment values for all delicate tissue variables in the symmetric gathering, including the upper incisor-sella-nasion plane angle (UI-SN), lower incisor mandibular plane angle (L-IMPA), and Frankfurtmandibular plane angle (FMA) plane points (p<0.05). The pre- and post-treatment values for FMA and L-IMPA altered significantly (p<0.05) in the uneven collection, but none of the fragile tissue boundaries did. When it came to UI-SN and FMA borders, as well as other delicate tissue variables other than Z, the SEP and asymmetric extraction patterns (AEP) groups were radically different. Deviated extractions, on the other hand, may be used to correct occlusal abnormalities without risking profile flattening, as is the case with symmetrical extraction designs. When using premolar extractions to lower the face level, careful consideration should be paid to biomechanics [21].

Class II area malocclusion therapy with unequal premolar extractions was the focus of a review headed by Janson et al., which aimed to examine the course of third molar angulation and its accessible space [21]. There were 37 patients in bunch 1 (type 1 class II development) and 25 in bunch 2 that took part in the trial (type 2 class II region). Extractions were performed in the class I mandibular quadrant and the two maxillary quadrants in lot 1. In the class II maxillary quadrant, extraction was performed in lot 2. Pre- and post-treatment panoramic radiographs were used to evaluate the angulation of the third molars and their accessible area. Results demonstrate that in type 1, third molar angulations and the area available on the extraction quadrants of the maxillary arch were both improved compared to type 2. After treatment, the extraction quadrant of the mandibular curve showed significantly greater improvement in angulation and accessibility to space. On the extraction quadrant, the angulation and accessible space for the maxillary third molar improved substantially more in type 2. A similar increase in available space for third molars was seen in the mandibular curve. When compared to the homologous non-extraction quadrants, the extraction quadrants after treatment had better angulation and a more noticeable space for third-molar ejections [25]. Differential treatment planning is an open, creative process. A variety of possibilities may arise. Adult patients were often present with mutilated or otherwise atypical dental problems, dictating unique approaches to treatment. Additionally, the goals of treatment might be quite specific. One constant, however, should always remain: the treatment result should be consistent with an aesthetic, stable, and healthy occlusion with pleasing facial harmony [26,27].

## Conclusions

The extraction of teeth as part of orthodontic therapy is only a tool and does not, in and of itself, constitute either a positive or negative action. When utilized improperly, they are capable of producing disastrous effects both functionally and aesthetically. However, when used appropriately, they make the treatment more stable and of higher quality. When planning an orthodontic tooth extraction, it is imperative that the width and length of the face be taken into consideration at all times. Oral hygiene, carious activity, periodontal involvement, the prognosis of impacted teeth, supernumeraries, and hypodontia are all factors that should be considered when developing an orthodontic treatment plan. The success of the treatment will depend on a comprehensive medical and dental history, as well as an extraoral and intraoral examination, diagnosis, and treatment planning that adhere to a systematic approach to treatment. According to the research examined, asymmetric extractions provide additional advantages for the patient, such as requiring less tooth structure to be removed. A thorough diagnostic evaluation makes accurate treatment outcomes possible. Always choose the extraction pattern based on the total health of the teeth, not simply on the simpler treatment biomechanics. For treatment stability, the inter-canine width has to be maintained or altered a little. In order to get the greatest potential outcomes in terms of both function and aesthetics, the treatment mechanics must be better understood and controlled in order to meet contemporary standards for face-centered therapy.
